# Supplementary material for: Increased Expression and Activation of FAK in Small-Cell Lung Cancer Compared to Non-Small-Cell Lung Cancer
Source: Cancers (Basel). 2019 Oct 10;11(10):1526. doi: 10.3390/cancers11101526 (PMC6827365; doi:10.3390/cancers11101526)
Supplement: Supplementary file 1 [file cancers-11-01526-s001.pdf]

Supplementary Materials: Increased Expression and Activation of FAK in Small-Cell Lung Cancer Compared to Non-Small-Cell Lung Cancer

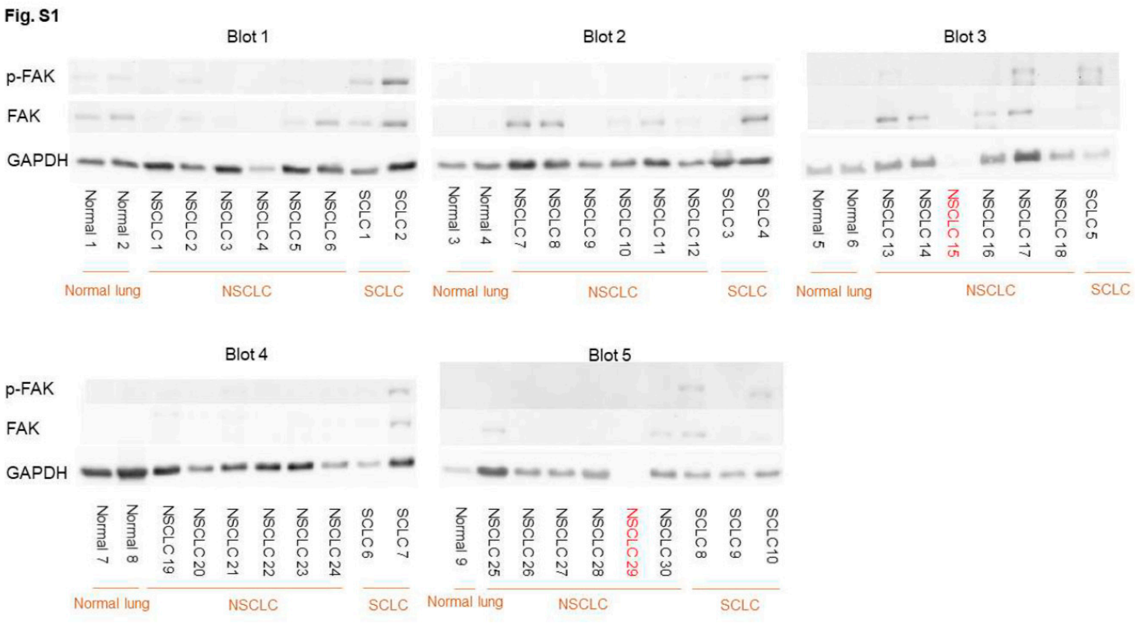

Figure S1. Western blot.
